# Supplementary material for: Conservation of dichromatin organization along regional centromeres
Source: Cell Genom. 2025 Mar 26;5(4):100819. doi: 10.1016/j.xgen.2025.100819 (PMC12008808; doi:10.1016/j.xgen.2025.100819)
Supplement: Document S1. Figures S1–S5 and Table S1 [file mmc1.pdf]

Cell Genomics, Volume *5*

Supplemental information

Conservation of dichromatin organization along regional centromeres

Danilo Dubocanin, Gabrielle A. Hartley, Adriana E. Sedeño Cortés, Yizi Mao, Sabrine Hedouin, Jane Ranchalis, Aman Agarwal, Glennis A. Logsdon, Katherine M. Munson, Taylor Real, Benjamin J. Mallory, Evan E. Eichler, Sue Biggins, Rachel J. O'Neill, and Andrew B. Stergachis

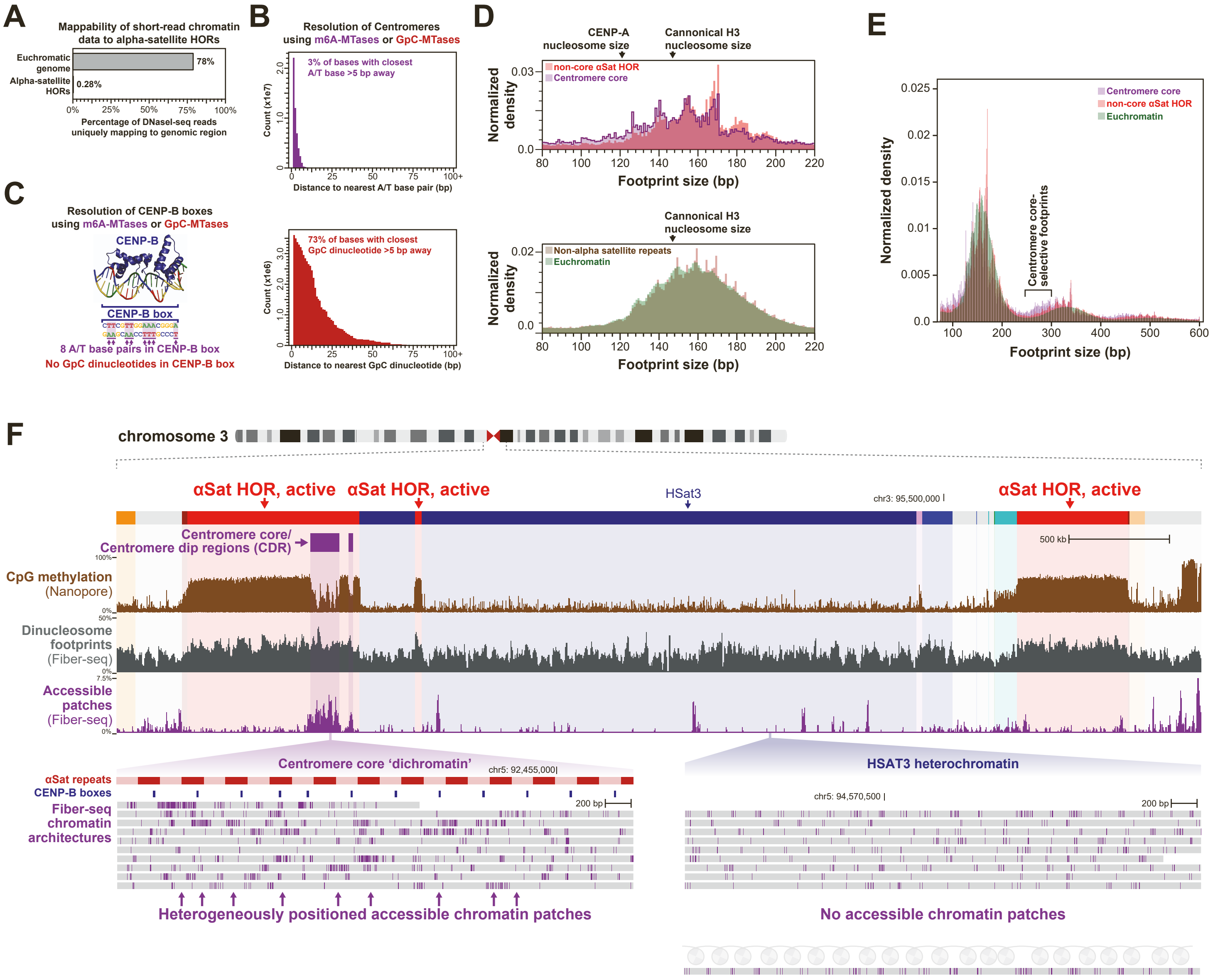

Figure S1. Single-molecule chromatin figure sequencing of human centromeres, related to Figure 2

(A) Bar plot showing the percentage of short-read DNaseI-seq data mapping to euchromatic genomic regions as well as alpha-satellite DNA that are uniquely mapping to these regions.

(B-C) Resolution of GpC methyltransferase-based and non-specific m6A-methyltransferase-based methods for mapping chromatin architectures within the centromere (B) as well as CENP-B occupancy along CENP-B boxes (C).

(D-E) Histograms of nucleosome footprint sizes within the centromere core, as well as in other genomic regions.

(F) Genomic locus of chromosome 3 centromere showing satellite repeats, bulk CpG methylation, Fiber-seq identified di-nucleosome footprint density, as well as Fiber-seq identified chromatin accessibility density. Below are individual Fiber-seq reads (grey bars) with m6A-modified bases in purple delineating single-molecule chromatin architectures within centromere core and HSat3 heterochromatic regions.

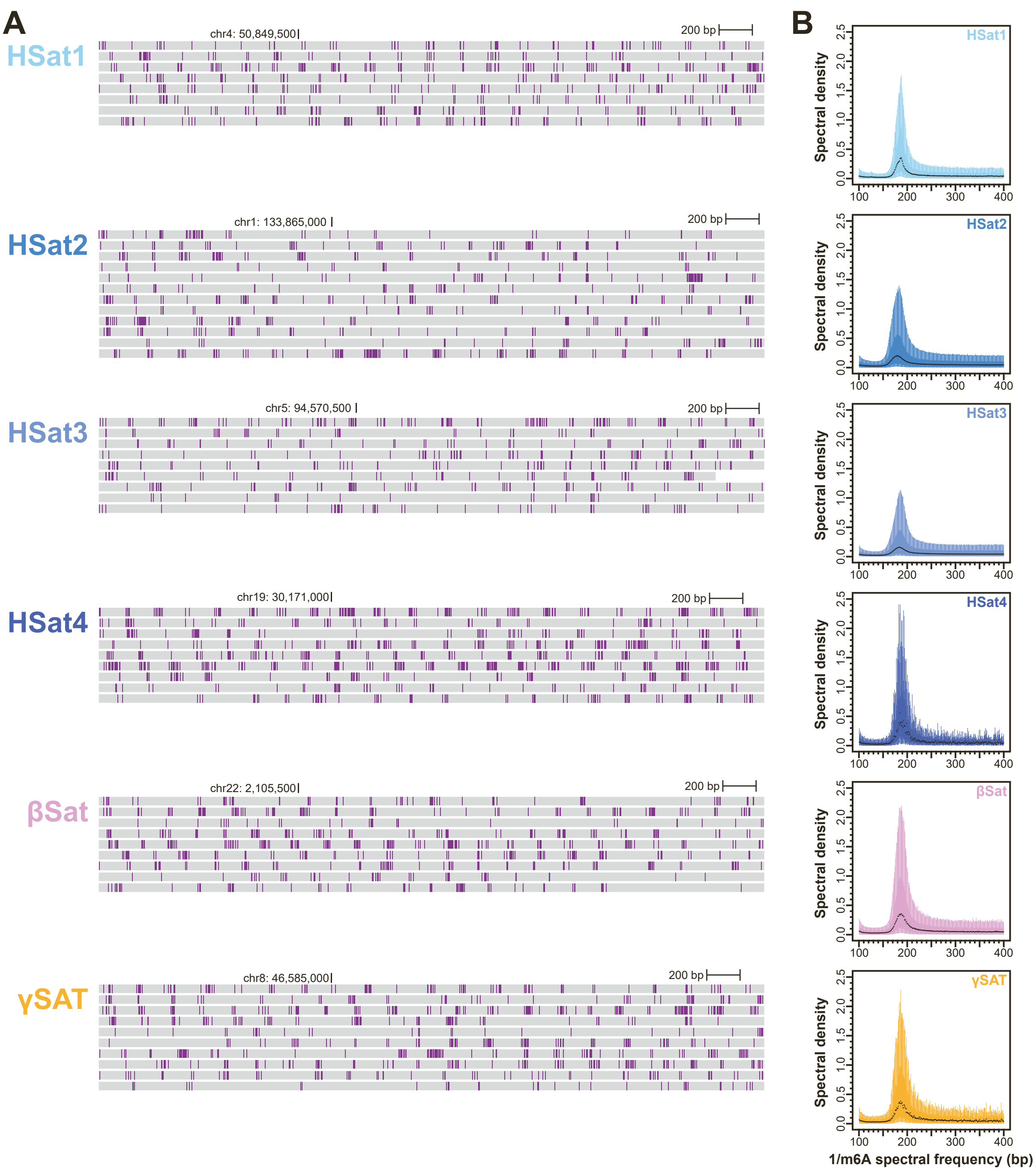

Figure S2. Higher-order chromatin architectures of heterochromatin, related to Figure 3
(A) Genomic loci showing single-molecule chromatin architectures within six separate satellite regions.
(B) Box-and-whisker plots of the chromatin repeat lengths from each of these six distinct satellite regions.

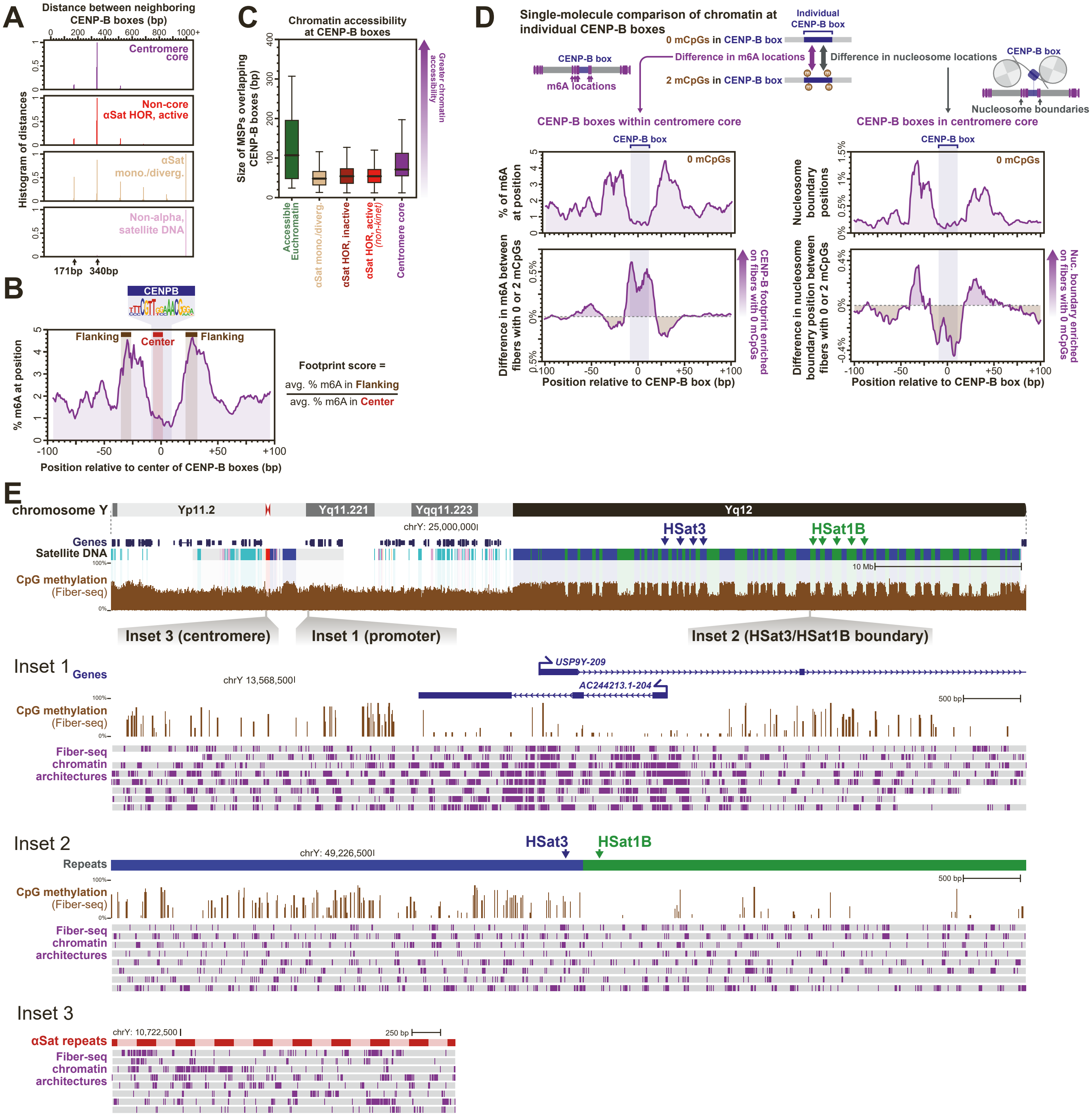

Figure S3. Single-molecule CENP-B occupancy within centromere core, related to Figure 4

(A) Histogram of the distance between CENP-B boxes within various genomic regions

(B) Aggregate m6A methylation profile at all CENP-B boxes located within the centromere core. Regions used for calculating footprint score are highlighted, and the calculation of the footprint score is enumerated below.

(C) Box-and-whisker plots showing the size of MSPs overlapping CENP-B boxes identified within different genomic regions.

(D) Directly comparing m6A-accessibility and nucleosome footprint boundaries at individual CENP-B boxes based on whether the read does or does not contain mCpG within that CENP-B box. Above is the aggregate m6A methylation (left) or nucleosome boundary positions (right) at centromere core alpha satellite CENP-B boxes containing no mCpG. Below is the aggregate difference in these features between reads with and without mCpG overlapping the CENP-B box.

(E) Genomic locus showing CpG methylation signal along the entire chromosome Y in HG002/GM24385 cells. Below are insets of single-molecule chromatin architectures at a promoter within the euchromatic region of chromosome Y, as well as an HSat3-HSat1B boundary within the heterochromatic q arm of chromosome Y.

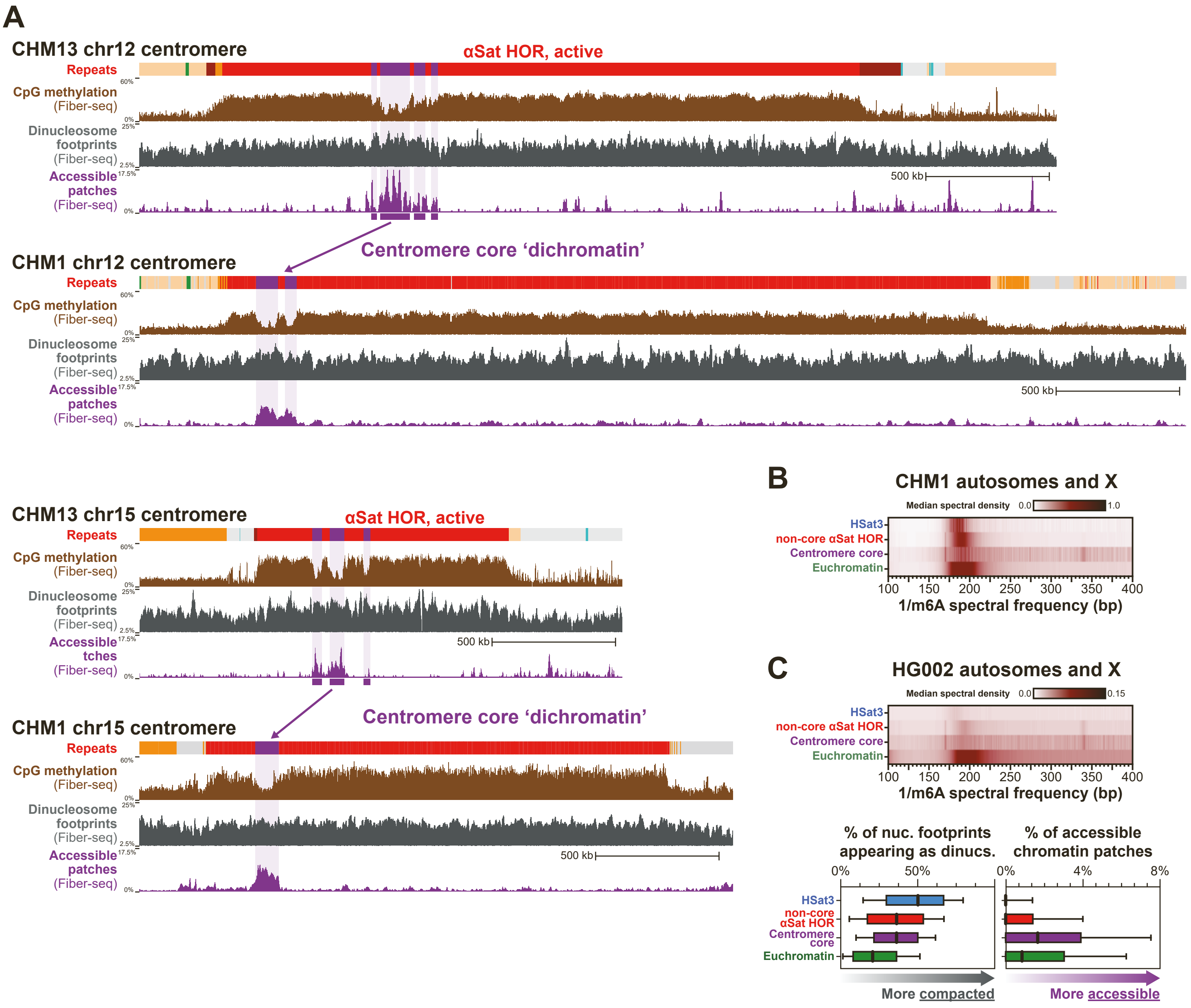

Figure S4. Conservation of dichromatin architecture between humans, related to Figure 5

(A) Genomic loci showing CpG methylation signal as well as di-nucleosome density and chromatin accessibility along the chromosome 12 and 15 centromeres in both CHM13 and CHM1 cells.

(B) Heatmap of the median spectral density for various genomic regions along the autosomes and X chromosome from CHM1 using Fiber-seq data from the CHM1 cell line.

(C) (top) Heatmap of the median spectral density for various genomic regions along the autosomes and X chromosome from HG002 using Fiber-seq data from the GM24385 cell line. (bottom) Average density of di-nucleosome footprints and accessible chromatin patches within various genomic regions along the autosomes and X chromosome from HG002 using Fiber-seq data from the GM24385 cell line.

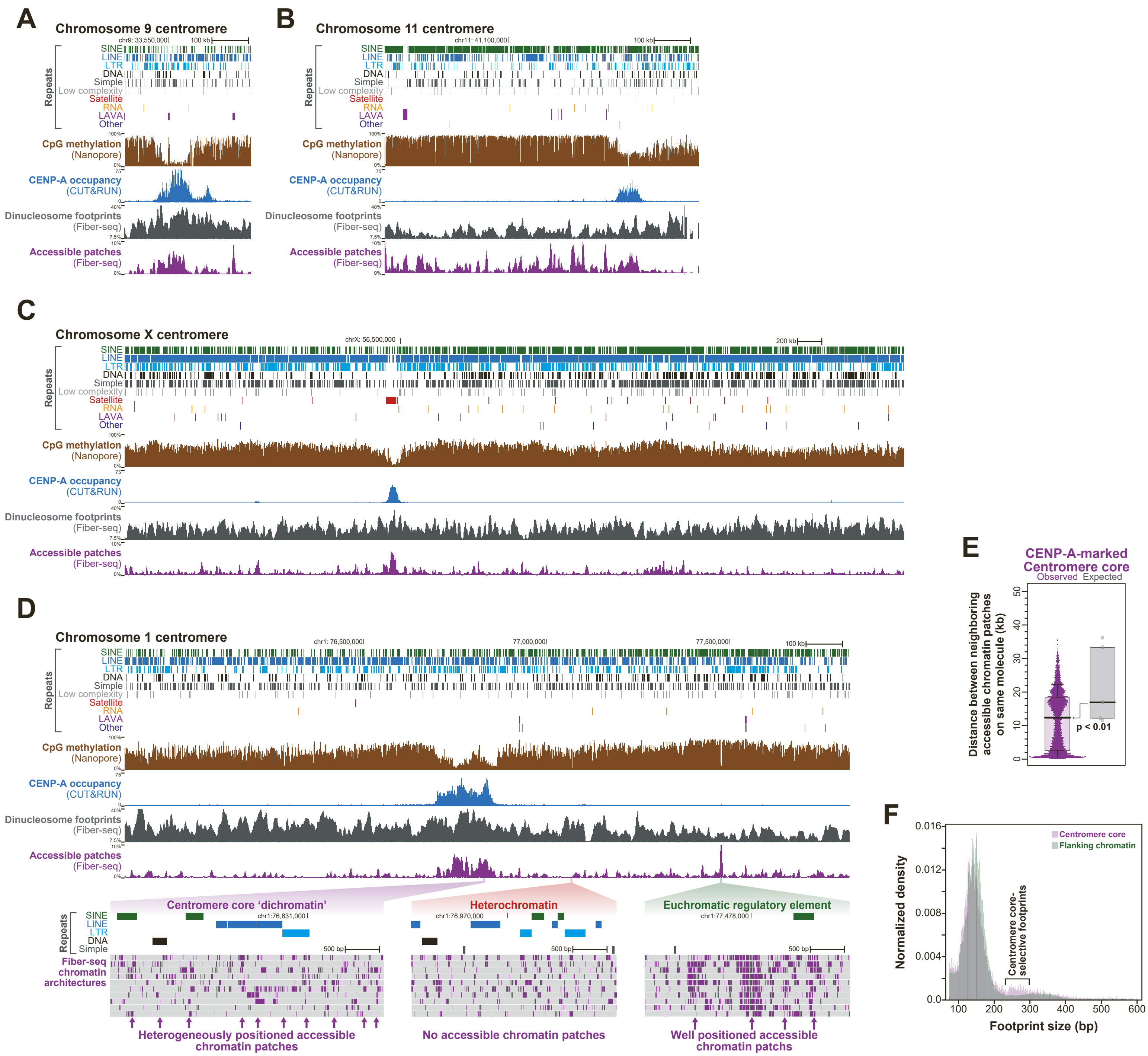

Figure S5. Alpha-satellite DNA is not necessary for dichromatin formation, related to Figure 6

(A-D) Genomic loci showing repeats, CpG methylation, CENP-A CUT&RUN, and Fiber-seq derived di-nucleosome density and chromatin accessibility along four assembled centromeres from a lymphoblastoid cell line from the eastern hoolock gibbon (Hoolock leuconedys) Betty. Below inset showing single-molecule chromatin architectures within the centromere core region, a heterochromatic region, and a gene regulatory element along chromosome 1.

(E) Swarm and box-and-whisker plots showing the distance between accessible chromatin patches along the same molecule of DNA within the centromere core, as well as the expected distance based on the density of accessible chromatin patches within each chromosome's centromere core (* p-value <0.01 Mann-Whitney).

(F) Histogram of nucleosome footprint sizes within the centromere core, as well as flanking genomic regions.

Sample	Genomic Read Depth	% Bases Covered	Centromeric Read Depth *	% Centromere Bases Covered*	Mean Read Length
CHM13	25.17	94.19%	28.66	98.9	17,761
CHM1	12.76	97.08%	14.30	99.29	22,185
HG002 *	13.37	99.47%	12.05	100	14,992
HLE	12.23	99.84%	11.99	100	19,769

* No q40 filter

Table S1. Sequencing statistics, related to Figure 2.
Sequencing statistics for the four human/primate samples used in this study
